# KLF9 positively regulates TRIM33 to inhibit abnormal synovial fibroblast proliferation, migration as well as inflammation in rheumatoid arthritis

**DOI:** 10.1002/iid3.696

**Published:** 2022-10-26

**Authors:** Dan Huang, Liju Tao, Xiuri Du

**Affiliations:** ^1^ Department of Rheumatology and Immunology Affiliated Hospital of Youjiang Medical College for Nationalities Baise Guangxi Zhuang Autonomous Region China

**Keywords:** KLF9, rheumatoid arthritis, synovial fibroblasts, TRIM33

## Abstract

**Background:**

Rheumatoid arthritis (RA) can cause irreversible joint injury and serious disability. This study aimed to investigate how TRIM33 regulated by KLF9 affects the aggressive behaviors of synovial fibroblasts induced by tumor necrosis factor‐α (TNF‐α).

**Materials and Methods:**

TNF‐α‐induced MH7A cells were used to simulate the in vitro model of RA. TRIM33 and KLF9 expression in TNF‐α‐challenged MH7A cells and transfection efficiency were analyzed via real‐time reverse transcription polymerase chain reaction together with western blot. The viability, proliferation, invasion, and migration of TNF‐α‐induced MH7A cells after transfection was respectively detected by CCK‐8, EdU staining, transwell, and wound‐healing assays. The expression of invasion and migration‐related proteins and inflammation‐related proteins was determined by western blot and the levels of inflammatory factors were detected by enzyme‐linked immunosorbent assay. The combination between TRIM33 and KLF9 was substantiated through dual‐luciferase reporter assay and chromatin immunoprecipitation.

**Results:**

TRIM33 and KLF9 expression in TNF‐α‐challenged MH7A cells was downregulated. TRIM33 elevation inhibited TNF‐α‐elicited proliferation, metastasis as well as inflammation of MH7A cells. Moreover, KLF9 was combined with TRIM33 and KLF9 promoted transcription of TRIM33. The inhibitory effect of TRIM33 overexpression on proliferation, invasion and migration and inflammation of MH7A cells induced by TNF‐α was alleviated by the downregulation of KLF9.

**Conclusion:**

KLF9 positively regulates TRIM33 to suppress the abnormal MH7A cell proliferation, migration, and reduce inflammation upon exposure to TNF‐α, which was reversed by inhibiting KLF9.

## Graphical abstract

KLF9 positively regulates TRIM33 to inhibit the abnormal proliferation and migration and reduce inflammation of TNF‐a‐induced MH7A cells, which was reversed by inhibiting KLF9.

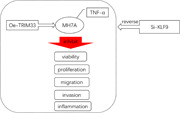

## 1 INTRODUCTION

Rheumatoid arthritis (RA) is one of the most prevalent diseases, affecting 0.5%–1% of the global population, which greatly affects people's quality of life.^1^ RA is a chronic autoimmune disorder featuring synovial dysplasia, synovitis as well as soft destruction of joints. Excessive proliferation and inadequate apoptosis of fibroblast‐like synoviocytes (FLSs) and infiltration of inflammatory factors are important causes of RA.^2^ At present, there is still a lack of effective drugs to intervene and reverse the destruction of bone in RA patients.

Tripartite motif‐containing 33 (TRIM33) belongs to TRIM family containing E3 ubiquitin ligases, whose members are involved in multiple pivotal biological events.^3^, ^4^, ^5^, ^6^ After stimulation by angiotensinogen II (Ang II), the expression of TRIM33 was specifically fortified in primary cardiac fibroblasts and myocardial tissues of newborn mice. Overexpression of TRIM33 inhibited the fibrosis induced by Ang II in mice and accelerated cardiac repair, functional recovery, and restricted cardiac fibrosis.^7^ TRIM33 was overexpressed in alveolar macrophages and fibroblasts in idiopathic pulmonary fibrosis patients and fibrotic lungs of rodents. TRIM33 gene knockout made mice sensitive to bleomycin (BLM)‐induced fibrosis, and AdCre‐TRIM33 inhibition aggravated BLM‐induced pulmonary fibrosis in mice.^8^ Therefore, TRIM33 may defend against fibrosis. TRIM33 suppressed the oxidative stress‐induced apoptosis of osteoblasts by inhibition of FOXO3a ubiquitination and degradation.^9^ The expression of TRIM33 increased during osteoblast differentiation, and TRIM33 promoted osteoblast proliferation and differentiation through BMP pathway.^10^ It is speculated that TRIM33 may have a certain protective effect on bone.

Via HumanTFDB (http://bioinfo.life.hust.edu.cn/HumanTFDB#!/), the binding of the transcription factor KLF9 to the TRIM33 promoter is predicted. KLF9 upregulation inhibited tumor necrosis factor‐α (TNF‐α)‐evoked human FLS migration, proliferation as well as inflammation.^11^ KLF9 protein expression was significantly downregulated in RA synovial fibroblasts (RASF) relative to healthy synovial fibroblasts, and elevated KLF9 expression suppressed RA development.^12^

Therefore, we hypothesized that KLF9 positively regulated the transcription of TRIM33, which inhibited RA progression.

## 2 MATERIALS AND METHODS

### 2.1 Cell culture and TNF‐α induction

Human MH7A cells were brought from Mingzhou Biotechnology Co., Ltd. and maintained in Dulbecco's modified Eagle's medium (DMEM) added with 10% fetal bovine serum (FBS), 100 U/ml penicillin, and 100 μg/ml streptomycin in a humidified atmosphere with 5% CO_2_ at 37°C. When cells reached to 70%–80% confluence, 24 h of treatment with TNF‐α (10 ng/ml; Sigma‐Aldrich) in MH7A cells in the six‐well plates (2 × 10^6^ cells/well) was implemented.^13^

### 2.2 Cell transfection

Guangzhou Ribobio Co., Ltd. synthesized ovexpression‐TRIM33 (Oe‐TRIM33) and its control (Oe‐NC), KLF9‐specific siRNA (Si‐KLF9) and Si‐NC. MH7A cells in six‐well plates (2 × 10^6^ cells/well) were transduced with Oe‐TRIM33, Oe‐NC, Si‐KLF9, and Si‐NC employing lipofectamine® 3000 (Invitrogen) for 48 h, and the transduction efficacy was determined. The transfected cells were continuously induced by TNF‐α (10 ng/ml; Sigma‐Aldrich) for 24 h.

### 2.3 Real‐time reverse transcriptase‐polymerase chain reaction (RT‐qPCR)

After transfection or TNF‐α induction, total RNA from MH7A cells was obtained by utilizing Trizol (Takara). The obtained cDNA applying Thermo Reverse Transcription kits (Western Biotech. Co., Ltd) was subjected to amplification utilizing Quanti Nova SYBR Green PCR kit (Qiagen GmbH) in the ABI 7300 system (ABI). The 2^–ΔΔCT^ method was adopted to quantify relative TRIM33 and KLF9 expression against GAPDH. The primer sequences are as follows: TRIM33, forward 5′‐TGACATCACAGGCCTTTCCC‐3′ and reverse 5′‐AGCATCTTCCAACTGTATGGGT‐3′;

KLF9, forward 5′‐CGTCAGTGGACTCGGGAAAA‐3′ and reverse 5′‐TGGACTTTCCAGACTGTCGC‐3′;

GAPDH, forward 5′‐ATGGGCAGCCGTTAGGAAAG‐3′ and reverse 5′‐AGGAAAAGCATCACCCGGAG‐3′.

### 2.4 Western blot analysis

After transfection or TNF‐α induction, cell lysates of MH7A cells were acquired via RIPA lysis buffer. Twelve percent SDS‐PAGE gel‐separated equal amounts of protein samples (60 μg) were electroblotted onto a PVDF membrane which was then impeded by 5% skim milk. The membranes were then maintained with TRIM33 (ab300146; dilution, 1:1000; Abcam), MMP‐1 (ab134184; dilution, 1:1000; Abcam), MMP‐3 (ab52915; dilution, 1:1000; Abcam), MMP‐9 (ab76003; dilution, 1:1000; Abcam), iNOS (ab178945; dilution, 1:1000; Abcam), COX‐2 (ab179800; dilution, 1:1000; Abcam), KLF9 (ab227920; dilution, 1:1000; Abcam), and GAPDH (ab9485; dilution, 1:2500; Abcam) primary antibodies overnight at 4°C. After TBST washing, 1 h of cultivation of membranes with HRP‐conjugated secondary antibody (ab6721; dilution, 1:2000; Abcam) was conducted at room temperature. Finally, ImageJ 1.8.0 Software evaluated the immunoreactive bands treated with ECL reagents.

### 2.5 CCK‐8 assay

After transfection or TNF‐α induction, the addition of CCK‐8 reagent to each well where MH7A cells (5 × 10^3^ cells/well) were plated in and incubated lasted for 2 h. OD450 nm value was examined with a Microplate Reader (Thermo Fisher).

### 2.6 EdU staining

After transfection or TNF‐α induction, MH7A cells (1 × 10^5^ cells/well) inoculated in a 96‐well plate were cultivated for 2 h with 10 µM EdU (Thermo Fisher Scientific, Inc.). MH7A cells were stained with DAPI for 10 min at room temperature to show DNA (blue). The proliferation of MH7A cells was analyzed under a fluorescent microscope.

### 2.7 Wound‐healing assay

After transfection or TNF‐α induction, MH7A cells were plated in a six‐well plate (2 × 10^5^ cells/well) and cultured until the confluence reached to 90%. The cell monolayer was scratched using a 100 μL pipette tip and cultured for 24 h. Pictures of the scratches were taken at 0 and 24 h under a light microscope.

### 2.8 Transwell assay

After transfection or TNF‐α induction, MH7A cells were seeded in the upper chamber precoated with Matrigel of a six‐well plate (2 × 10^6^ cells/well). The upper chamber was loaded by serum‐starved DMEM medium and the bottom chamber was supplemented by the complete medium. The noninvasive cells were removed 24 h later and the invasive cells were stained with 0.1% crystal violet (Solarbio) at room temperature for 10 min. Finally, cells were photographed under a light microscope.

### 2.9 Enzyme‐linked immunosorbent assay (ELISA)

After transfection or TNF‐α induction, culture supernatants of MH7A cells were collected and the contents of inflammatory factors were separately determined via IL‐1β (cat. no. PI305), IL‐6 (cat. no. PI330), IL‐8 (cat. no. PI640) ELISA kits (Beyotime) following the manufacturer's instructions.

### 2.10 Dual‐Luciferase Reporter Assay

The wild‐type (WT) and mutant (MUT) sequences of TRIM33 were cloned into a pGL3 luciferase vector (Promega Corporation) and respectively named TRIM33‐WT and TRIM33‐MUT. MH7A cells were cotransfected with TRIM33‐WT or TRIM33‐MUT and Si‐KLF9 or Si‐NC using lipofectamine® 3000 (Invitrogen) and incubated for 48 h at 37°C. Finally, dual‐luciferase reporter assay kit (Beyotime) was to test the firefly luciferase activities.

### 2.11 Chromatin immunoprecipitation (ChIP)

ChIP was conducted using the ChIP Assay Kit (cat. no. P2078; Beyotime) in light of the manufacturer's protocol. The chromatin–protein complexes were probed with KLF9 (cat. no. 5H16L7; Thermo Fisher Scientific) or IgG antibodies. Immunoprecipitated DNA was analyzed via RT‐qPCR.

### 2.12 Statistical analysis

The experimental data analyzed by GraphPad Prism software were presented as mean ± SD (SD). *P* value among multiple groups was used by one‐way ANOVA followed by Tukey's post hoc test and *p* value between two groups was performed by Student's *t‐*test. *p* < .05 was taken as statistically significant.

## 3 RESULTS

### 3.1 Overexpression of TRIM33 inhibits TNF‐α‐stimulated MH7A cell proliferation

**Figure 1:**
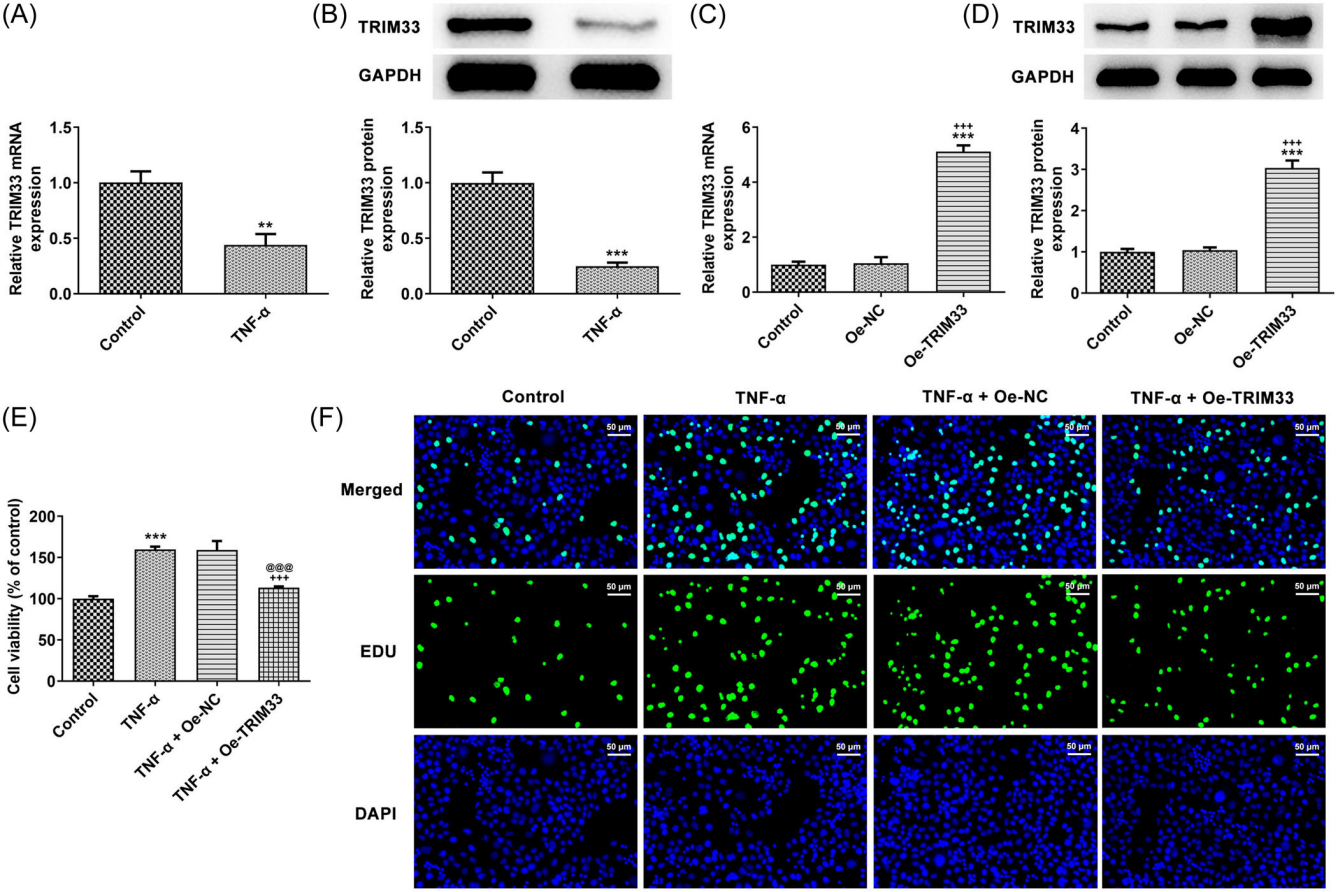
Overexpression of TRIM33 inhibits tumor necrosis factor‐α (TNF‐α)‐stimulated MH7A cell proliferation. (A, B) Real‐time reverse transcription polymerase chain reaction (RT‐qPCR) and western blot tested TRIM33 expression. **p <0.01 and ***p< 0.001 versus Control group. (C, D) RT‐qPCR and western blot tested overexpression efficacy of TRIM33. ***p< 0.001 versus Control group. ^+++^ *p <* 0.001 versus Oe‐NC group. The viability (E) and proliferation (F) of TNF‐α‐challenged MH7A cells were judged via CCK‐8 assay and EdU staining (magnification ×200). ***p < .001 versus Control group. ^+++^ *p* < .001 versus TNF‐α group. p@@@ < .001 versus TNF‐α+Oe‐NC group.

TRIM33 expression was decreased in MH7A cells challenged with TNF‐α (Figure 1A,B). The expression of TRIM33 was upregulated in MH7A cells transfected with Oe‐TRIM33 (Figure 1C,D). The viability and proliferation of MH7A cells were promoted by TNF‐α and overexpression of TRIM33 inhibited the viability and proliferation of TNF‐α‐insulted MH7A cells (Figure 1E,F).

### 3.2 TRIM33 elevation inhibited TNF‐α‐triggered MH7A cell metastasis

**Figure 2:**
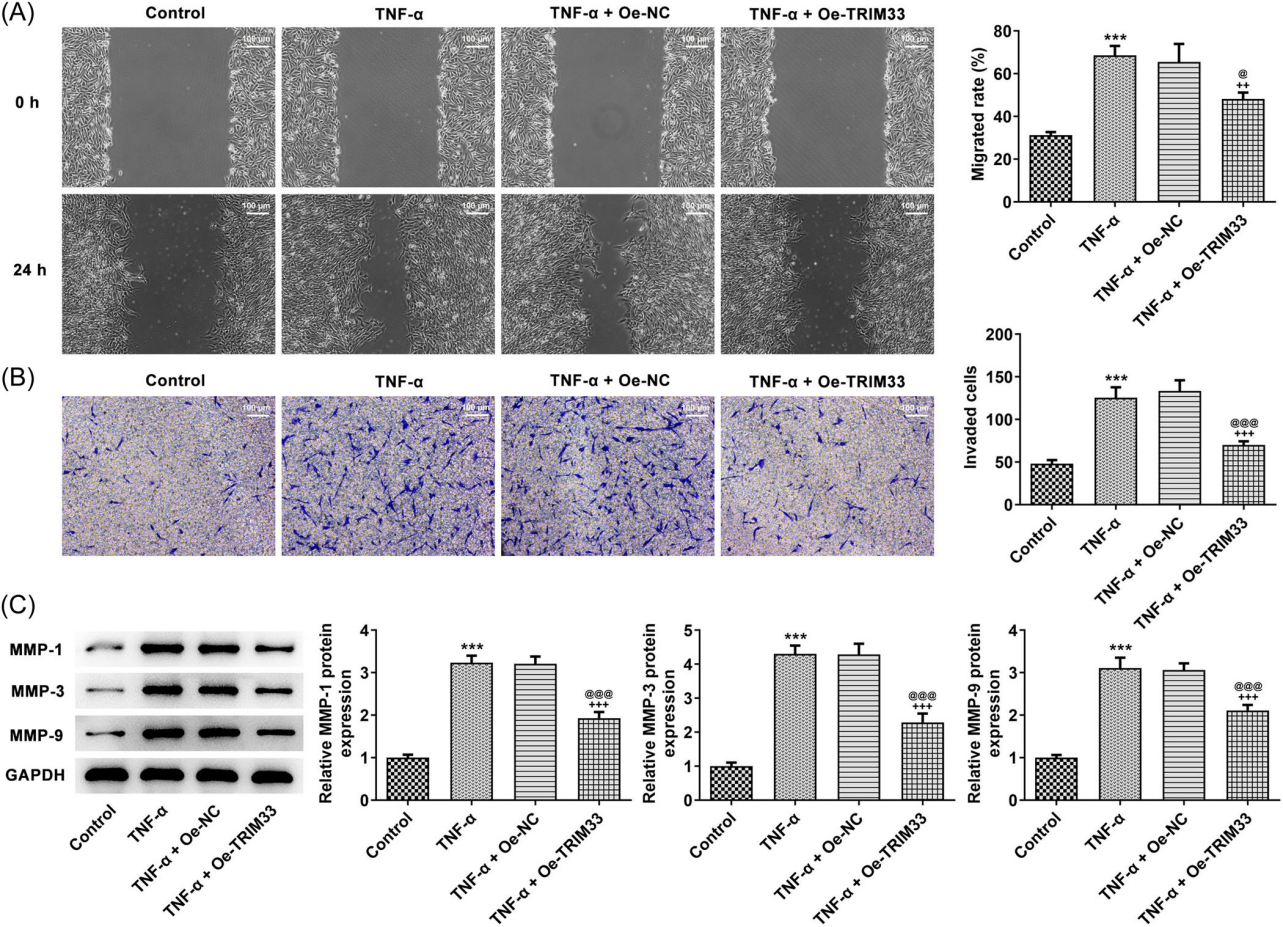
TRIM33 elevation inhibited TNF‐α‐triggered MH7A cell metastasis. The migration (A) and invasion (B) of TNF‐α‐insulted MH7A cells were appraised via transwell and wound‐healing assays (magnification ×100). (C) Western blot tested the expression of metastasis‐related proteins. ***p < .001 versus Control group. ^++^ *p* < .01 and ^+++^ *p* < .001 versus TNF‐α group. ^@^ *p* < .05 and p@@@ < .001 versus TNF‐^α+^Oe‐NC group.

TNF‐α enhanced MH7A cell metastasis, which was suppressed following TRIM33 elevation (Figure 2A,B). MMP‐1, MMP‐3, and MMP‐9 expression was upregulated in TNF‐α‐exposed MH7A cells and also these proteins expression induced by TNF‐α could be inhibited by the overexpression of TRIM33 (Figure 2C).

### 3.3 Overexpression of TRIM33 attenuates TNF‐α‐elicited MH7A cell inflammation

**Figure 3:**
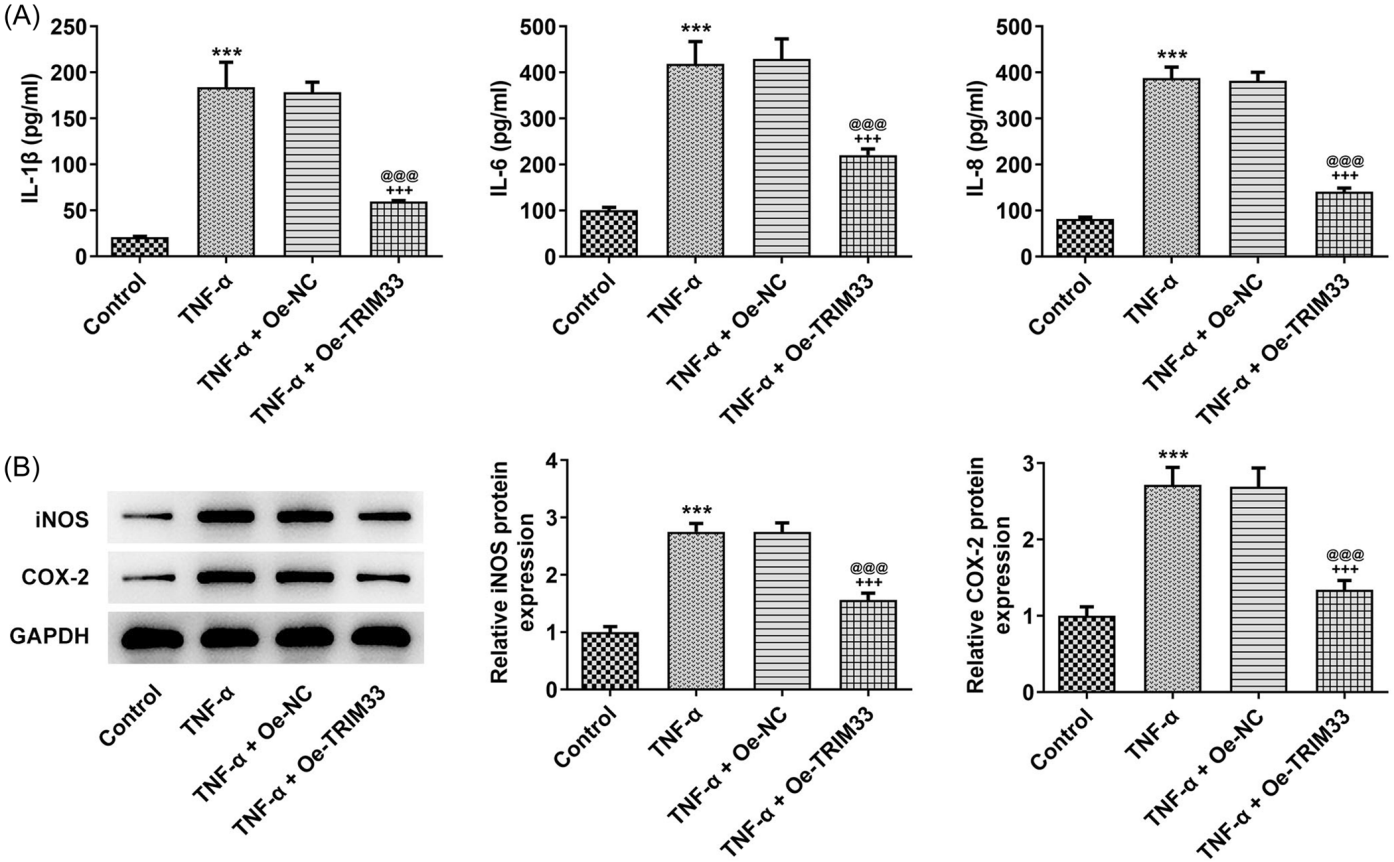
Overexpression of TRIM33 attenuates TNF‐α‐elicited MH7A cell inflammation. (A) interleukin‐1β (IL‐1β), IL‐6, and IL‐8 levels were examined with respective ELISA kits. (B) Western blot tested inflammation‐associated protein expression. ***p < .001 versus Control group. ^+++^ *p* < .001 versus TNF‐α group. p@@@ < .001 versus TNF‐α^+^Oe‐NC group.

Interleukin‐1β (IL‐1β), IL‐6, and IL‐8 levels in MH7A cells exposed to TNF‐α were increased and overexpression of TRIM33 downregulated the above pro‐inflammatory factors levels (Figure 3A). iNOS and COX‐2 expression in TNF‐α‐challenged MH7A cells was increased, which was reversed by the overexpression of TRIM33 (Figure 3B).

### 3.4 KLF9 promotes transcription of TRIM33

**Figure 4:**
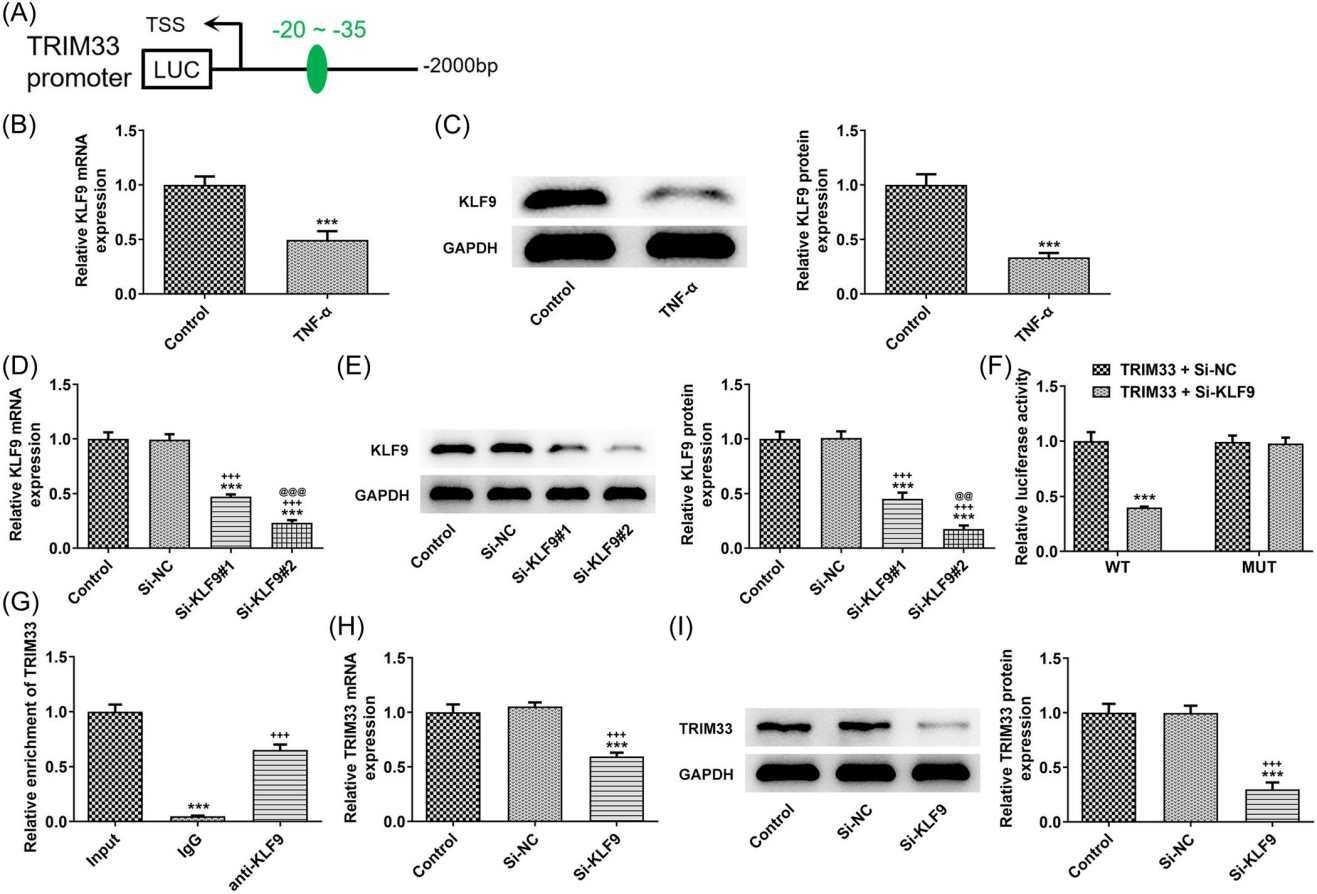
KLF9 promotes transcription of TRIM33. (A) HumanTFDB predicted binding sites for transcription factors KLF9 and TRIM33 promoter. (B, C) Real‐time reverse transcription polymerase chain reaction (RT‐qPCR) and western blot tested KLF9 expression. ^***^ *p <*0.001 versus Control group. (D, E) RT‐qPCR and western blot tested the interference efficacy of KLF9. ***p < 0.001 versus Control group. ^+++^ *p <* 0.001 versus Si‐NC group. p@@ *<*0.01 and p@@@ *<* 0.001 versus Si‐KLF9#1 group. (F) Dual‐luciferase reporter assay appraised TRIM33‐WT and TRIM33‐MUT luciferase activities. ***p < 0.001 versus TRIM33 + Si‐NC group. (G) Chromatin immunoprecipitation tested TRIM33 abundance in anti‐KLF9. ^***^ *p <* 0.001 versus Input group. ^+++^ *p <* 0.001 versus IgG group. (H, I) RT‐qPCR and western blot tested TRIM33 expression. ***p < .001 versus Control group. ^+++^ *p* < .001 versus Si‐NC group.

The binding sites between KLF9 and TRIM33 are shown in Figure 4A. The expression of KLF9 in MH7A cells was decreased by TNF‐α induction (Figure 4B,C). Following the transduction of KLF7 interference plasmids, KLF9 expression was decreased, and lower expression of KLF9 was observed in the  Si‐KLF9#2 group (Figure 4D,E). The luciferase activity was decreased in MH7A cells transduced by TRIM33‐WT and Si‐KLF9 (Figure 4F). The enrichment of TRIM33 was observed with the addition of anti‐KLF9 (Figure 4G). TRIM33 expression was decreased on account of KLF9 absence (Figure 4H,I).

### 3.5 KLF9 positively regulates TRIM33 and inhibits TNF‐α‐elicited proliferation, metastasis, and alleviates inflammatory responses in MH7A cells

**Figure 5:**
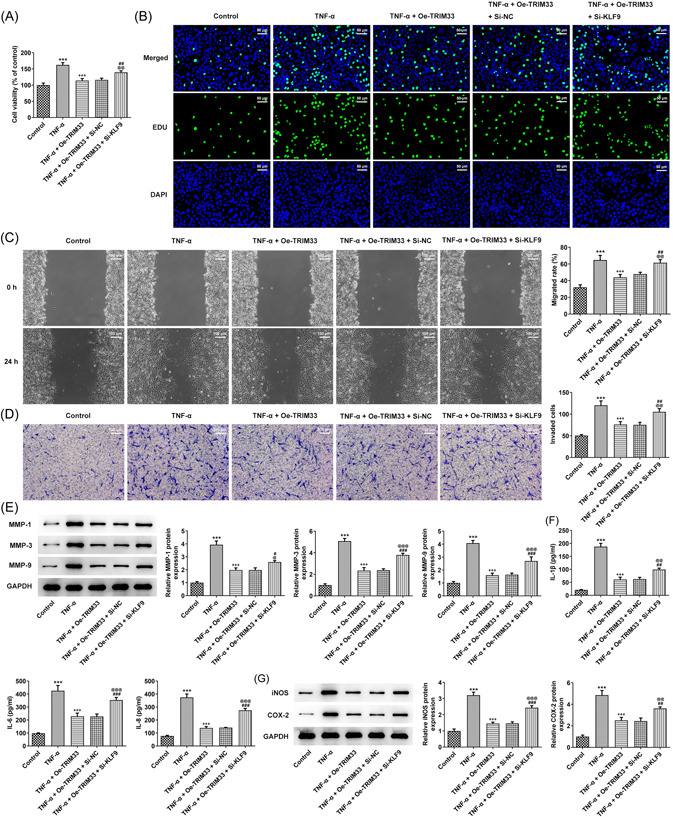
KLF9 positively regulates TRIM33 and inhibits tumor necrosis factor‐α (TNF‐α)‐elicited proliferation, metastasis, and alleviates inflammatory responses in MH7A cells. The viability (A) and proliferation (B) of TNF‐α‐challenged MH7A cells were judged via CCK‐8 assay and EdU staining (magnification ×200). The migration (C) and invasion (D) of TNF‐α‐insulted MH7A cells were appraised via transwell and wound‐healing assays (magnification ×100). (E) Western blot tested the expression of metastasis‐related proteins. (F) Interleukin‐1β (IL‐1β), IL‐6, and IL‐8 levels were examined with respective ELISA kits. (G) Western blot tested inflammation‐associated protein expression. ***p < .001 versus Control group. ^+++^ *p* < .001 versus TNF‐α group. ^@^ *p* < .05, p@@ < .01 and p@@@ < .001 versus TNF‐^α+^Oe‐TRIM33 group. ^#^ *p* < .05,^##^ *p* < .01, and ^###^ *p* < .001 versus TNF‐α + Oe‐TRIM33 + Si^‐^NC group.

TRIM33 elevation suppressed the viability and proliferation of TNF‐α‐induced MH7A cells, which was promoted by the downregulation of KLF9 (Figure 5A,B). The invasion and migration of TNF‐α‐challenged MH7A cells was diminished by TRIM33 overexpression and the inhibiting effect of TRIM33 overexpression was weakened by the downregulation of KLF9 (Figure 5C,D). As shown in Figure 5E, MMP‐1, MMP‐3, and MMP‐9 expression (Figure 5E), levels of IL‐1β, IL‐6, and IL‐8 (Figure 5F), and iNOS and COX‐2 expression (Figure 5G) were downregulated in the TNF‐α + Oe‐TRIM33 group comparted with that in the TNF‐α group, which was suppressed by the downregulation of KLF9.

## 4 DISCUSSION

RA is a systemic autoimmune disease characterized by synovial dysplasia and migration of synovial fibroblasts, resulting in erosion of cartilage and bone, and leading to joint destruction and dysfunction.^13^, ^14^, ^15^ During the pathogenesis of RA, various inflammatory cells such as T cells, B cells, and macrophages are activated to release inflammatory factors to stimulate the activation of synovial fibroblasts, leading to the increase of synovial fibroblasts proliferation and migration.^16^ Abundant synovial fibroblast proliferation belongs to the key characteristics of RA and proliferative synovial fibroblasts gradually erodes cartilage and bone, eventually leading to joint function destruction.^17^ Most importantly, proliferative synovial fibroblasts may promote the recruitment, retention, and infiltration of inflammatory cells such as macrophages and lymphocytes,^16^, ^18^, ^19^, ^20^ and aggravate the inflammatory response of RA by secreting more inflammatory factors.^21^, ^22^ Activated synovial fibroblast migration into cartilage or bone occupies a vital position in the formation of RA synovial invasive pannus.^23^ Therefore, how to effectively inhibit the proliferation and migration of synovial fibroblasts and inflammation in RA is of great significance for understanding the underlying mechanism of RA.

Studies have shown that the dysregulation of TRIM protein expression can lead to a variety of diseases, such as tumors, neuropsychiatric diseases, immune diseases, and so on.^24^ Recent studies have found that TRIM33 gene is lowly expressed in liver cancer and chronic myelogenous leukemia,^25^ and knocking out TRIM33 gene in mice can lead to the occurrence of liver cancer in mice.^26^ TRIM33 expression is decreased in renal cell carcinoma, and overexpression of TRIM33 in renal cell carcinoma can affect the proliferation, migration, and invasion of cancer cells by inhibiting the Wnt/β‐catenin pathway.^27^ In vitro and in vivo studies have confirmed that TRIM33 plays an important role in valproic acid inhibiting the migration and invasion of prostate cancer cells.^28^ This study also demonstrated that TRIM3 expression was decreased in TNF‐α‐induced MH7A cells and overexpression of TRIM3 suppressed the proliferation, invasion, and migration of TNF‐α‐induced MH7A cells.

The present study also showed that TRIM33 could inhibit the secretion of inflammatory factors in synovial fibroblasts. J. Qi et al.^29^ reported that anti‐IL‐1β antibody could significantly alleviate RA symptoms. P. Ruscitti et al.^30^ reported that inhibition of IL‐1 secretion was an effective treatment for RA. Y. Yoshida et al. showed that anti‐IL‐6 receptor‐related drugs, either alone or in combination with other drugs, could effectively treat RA. The treatment effect of simvastatin on RA was involved in inhibition of inflammation.^31^ Here, in TNF‐α‐challenged MH7A cells, TRIM33 was downregulated, resulting in its reduced inhibitory effect on IL‐1β, IL‐6, and IL‐8 secretion.

KLF9 is a transcription factor related to differentiation, widely involved in neural development, B‐cell differentiation, proliferation, apoptosis, and other processes.^32^ KLF9 was reported to be a key factor of oxidative stress in cells, which may enhance cellular oxidative stress and promote tumorigenesis by upregulating intracellular reactive oxygen species.^33^ KLF9 mRNA transcription and protein levels were decreased in colorectal cancer, and total or partial loss of KLF9 could promote colon tumorigenesis.^34^ Previous studies hinted that KLF9 suppressed TNF‐α‐induced MH7A cells or RA synovial fibroblast inflammation, migration, and proliferation.^11^, ^12^ Our study exposed that KLF9 downregulation reversed the inhibiting impact of TRIM33 to promote the proliferation, metastasis, and inflammation of TNF‐α‐insulted MH7A cells.

To conclude, TRIM33 and KLF9 expression was decreased in TNF‐α‐challenged MH7A cells, and KLF9 positively regulated TRIM33 expression. TRIM33 overexpression inhibited TNF‐α‐induced MH7A cell proliferation, metastasis, and inflammation of MH7A cells, which was countervailed by KLF9 depletion. A few limitations in this study were that the obtained conclusion was demonstrated by cell experiment, lacking in vivo experiment, and there is no related signaling pathway explored in this study. Only one MH7A cell line has been used in this study and we will use RA synovial fibroblasts isolated by enzymatic digestion of the synovial tissues for our further study.

## AUTHOR CONTRIBUTIONS

Dan Huang designed the study and drafted the manuscript. All authors analyzed the data and the manuscript was critically revised by Xiuri Du. All authors have confirmed the authenticity of all the raw data and read and approved the final manuscript.

## CONFLICT OF INTEREST

The authors declare no conflict of interest.

## Data Availability

The datasets used and/or analyzed during the current study are available from the corresponding author on reasonable request.
